# A review on the use of coconut oil to mitigate methane emissions in ruminants: mechanisms of action and research progress

**DOI:** 10.3389/fvets.2026.1774322

**Published:** 2026-04-30

**Authors:** Lun Sun, Xin Wang, Yong Long, Xu Wang, Xinran Niu, Huijie Li, Nittaya Taethaisong, Weerada Meethip, Siwaporn Paengkoum, Pramote Paengkoum

**Affiliations:** 1 School of Animal Technology and Innovation, Institute of Agricultural Technology, Suranaree University of Technology, Nakhon Ratchasima, Thailand; 2 Guizhou University of Engineering Science, Bijie, China; 3 Program in Agriculture, Faculty of Science and Technology, Nakhon Ratchasima Rajabhat University, Nakhon Ratchasima, Thailand

**Keywords:** coconut oil, medium-chain fatty acids, methane mitigation, rumen fermentation, ruminants, sustainable ruminant production

## Abstract

Methane (CH_4_) generated during ruminal fermentation in ruminants is a major contributor to greenhouse gas emissions and represents a substantial loss of dietary energy. Therefore, mitigating enteric CH_4_ emissions through safe and efficient nutritional strategies is of considerable ecological and economic significance. Coconut oil (CO) has received increasing attention due to its distinctive fatty acid profile. Accumulating evidence indicates that the medium-chain fatty acids (MCFA) abundant in CO exert direct inhibitory effects on rumen protozoa and methanogenic archaea, thereby conferring strong antimethanogenic potential. However, the CH_4_ mitigation efficacy of CO is often accompanied by trade-offs related to rumen microbial ecology, animal productive performance, and the nutritional quality of animal-derived products, particularly at higher doses where fiber digestibility and dry matter intake (DMI) may decline. Consequently, a clearer definition of safe and effective inclusion levels across different ruminant species and production stages is needed. This review systematically summarizes the physicochemical properties and safety of CO, with particular emphasis on its mechanisms of action within the rumen. Furthermore, current application studies and future research prospects of CO in ruminant production are discussed, providing a scientific reference for its use in nutritional strategy to lower methane in ruminant systems.

## 1 Introduction

Greenhouse gas emissions have become a central focus in discussions on the drivers of global climate change. CH_4_ is the second most important greenhouse gas after carbon dioxide (CO_2_) and possesses a substantially higher energy content per molecule than CO_2_ (1). Moreover, CH_4_ has an atmospheric lifetime of approximately 12.4 years, during which it exerts a strong warming effect, making it a critical contributor to global warming (2). Ruminants efficiently utilize fibrous plant materials through microbial fermentation in the rumen, an anaerobic process that inevitably generates large quantities of CO_2_ and CH_4_ (3, 4). CH_4_ produced during ruminal fermentation is primarily expelled via eructation from the rumen headspace, whereas CH_4_ generated during hindgut fermentation is released mainly through respiration or flatulence (5). Collectively, CH_4_ emissions from ruminant livestock account for approximately 5% of total global greenhouse gas emissions (6). In addition to its environmental implications, enteric CH_4_ production represents a substantial loss of dietary energy, estimated to range from 2 to 12% of gross energy intake, thereby reducing feed efficiency and overall animal productivity (7, 8). Viewed this way, strategies aimed at mitigating CH_4_ emissions are not only ecologically imperative but also economically advantageous. Among the various approaches investigated, nutritional interventions have attracted considerable interest, with dietary lipids emerging as promising candidates due to their capacity to modulate the rumen microbial ecosystem and alter hydrogen (H_2_) utilization pathways (9, 10).

Coconut oil (CO) is a byproduct of coconut processing and is extracted from the endosperm of mature coconuts (11). It represents one of the most economically important vegetable oils in tropical regions (12). The most distinctive feature of CO is its unique fatty acid profile, which is dominated by MCFAs, accounting for approximately 50–60% of total fatty acids (13, 14). Among these, saturated fatty acids (SFA) comprise approximately 80–92%, with lauric acid (C12:0) being the most abundant (45–53%), followed by myristic acid (C14:0) at around 19% (15–17). The high degree of saturation and elevated MCFA content confer CO with strong oxidative stability (13) and reduce its susceptibility to ruminal biohydrogenation. As a result, CO has the potential to alter electron flow and H_2_ utilization pathways in the rumen, processes that are directly linked to methanogenesis (18, 19). Previous studies have demonstrated that CO and its unique fatty acid composition exert direct inhibitory and potentially toxic effects on rumen protozoa and methanogenic archaea, thereby exhibiting substantial potential to suppress CH_4_ production and modulate rumen fermentation, ultimately leading to reduced enteric CH_4_ emissions (20–23). Nevertheless, the overall effects of CO supplementation on other functional rumen microorganisms, nutrient digestibility, and animal productive performance remain controversial (24–26).

Therefore, this review aims to synthesize recent evidence by summarizing experimental findings and the application progress of CO in ruminant nutrition, while highlighting current research limitations and identifying future research directions. Such an integrated assessment is essential to fully exploit the CH_4_ mitigation potential of CO without compromising rumen function or livestock production efficiency.

## 2 Physicochemical properties of CO

**Figure 1:**
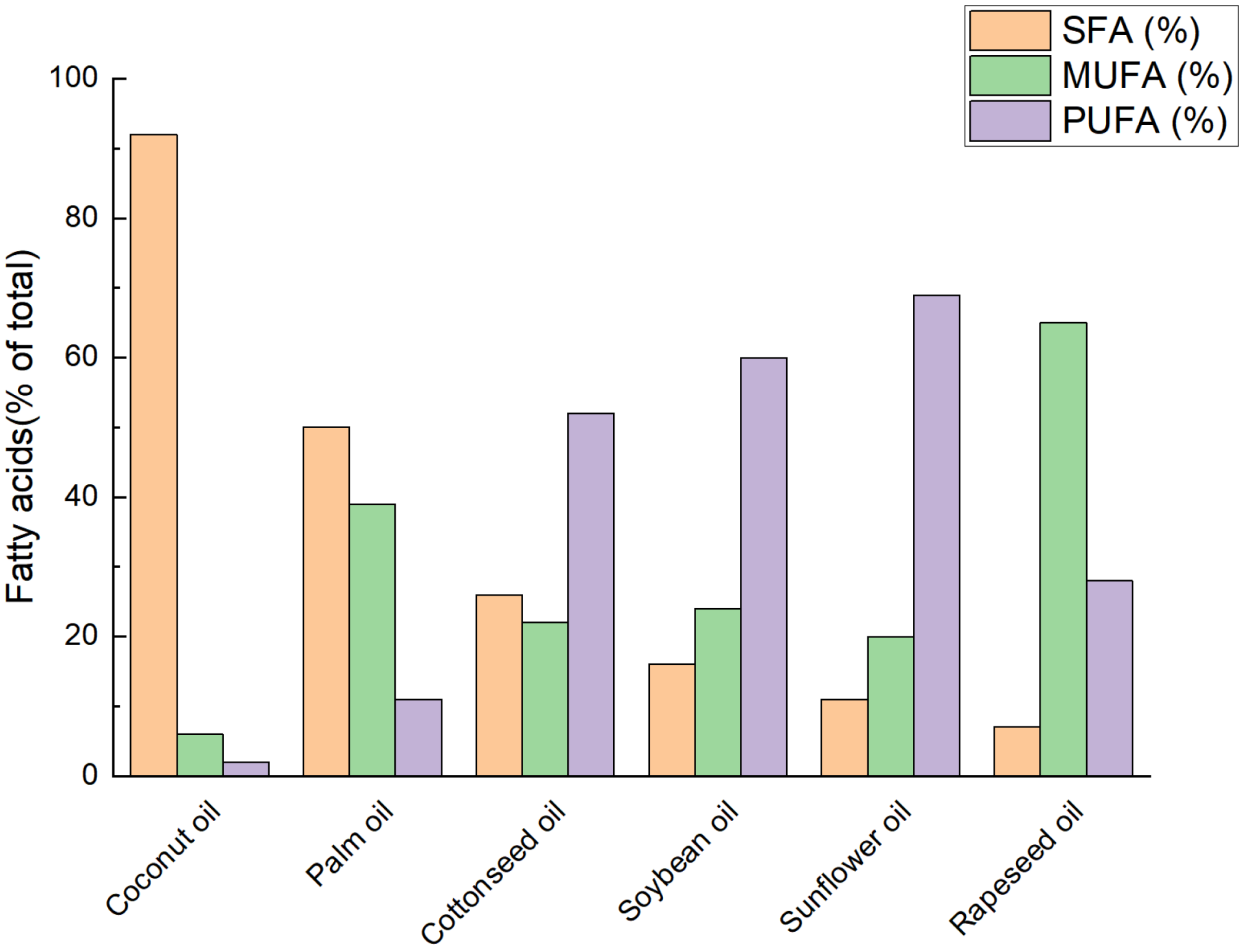
Typical fatty acid composition of CO compared with other common plant oils. Grouped bars represent the proportions of saturated (yellow), monounsaturated (green), and polyunsaturated (purple) fatty acids, expressed as a percentage of total fatty acids, in coconut, palm, cottonseed, soybean, sunflower, and rapeseed oils. The values shown are representative compositions compiled from published literature and standard reference tables and are intended to illustrate relative differences among oils rather than provide exact quantitative values (32, 41, 42). Accordingly, no statistical analysis was performed.

Coconut oil (CO) is typically a clear, colorless, or nearly colorless oily liquid. Owing to its relatively low melting point (approximately 23–26 °C), CO is often semi-solid at room temperature and can solidify into a white solid or creamy semi-solid at lower temperatures. This phase behavior is primarily attributable to its fatty acid composition, which is dominated by SFAs (27, 28). From a chemical classification perspective, CO belongs to the lauric oil group, together with palm kernel oil and babassu oil (29, 30). As illustrated in Figure 1, the fatty acid profile of CO differs markedly from that of other commonly used vegetable oils. Lauric acid (C12:0) is the predominant fatty acid in CO, accounting for nearly half of the total fatty acids, followed by myristic acid (C14:0, approximately 19%) and palmitic acid (C16:0, approximately 9%) (31, 32). The predominance of low-molecular-weight SFAs results in a high saponification value (approximately 250 mg KOH/g) and a low iodine value (approximately 10–20 g I₂/100 g) (17, 33). A high saponification value reflects a greater proportion of short- and medium-chain fatty acids, whereas a low iodine value indicates a low degree of unsaturation (34). Together, these parameters confirm the highly saturated nature of CO and help explain its excellent oxidative stability and extended shelf life (31).

From the standpoint of nutritional antioxidants, however, CO cannot be considered a major dietary source of antioxidant vitamins or polyphenols, as its total polyphenol content is extremely low (35, 36). Virgin coconut oil (VCO) has been reported to contain only about 38 mg/kg of total tocopherols (30), whereas refined CO, following bleaching and deodorization, contains negligible amounts of phenolic compounds and tocopherols (32). Nevertheless, an *in vivo* mouse study demonstrated that VCO supplementation at 10 mL/kg body weight exerted appreciable antioxidant effects, which implies that certain components of VCO may help mitigate oxidative damage (37). Similar findings have been reported in rats (38) and fish (39), and these effects have been attributed to the combined action of MCFAs and trace levels of bioactive minor compounds present in VCO. As shown in Figure 1, the fatty acid composition of CO is characterized by a substantially higher proportion of SFAs compared with conventional vegetable oils such as soybean, rapeseed, sunflower, and cottonseed oils, which typically contain only 7–26% SFAs (40–43). Accordingly, CO contains extremely low levels of unsaturated fatty acids (UFA), generally below 10%, mainly in the form of oleic acid (C18:1) (31, 123), further contributing to its oxidative stability.

In addition to its physicochemical characteristics, the digestion and absorption of fatty acids derived from CO differ markedly from those of long-chain fatty acids (LCFA) (44). Medium-chain triglycerides, owing to their shorter carbon chain length and greater hydrophilicity, are more readily hydrolyzed by gastric and pancreatic lipases and exhibit a much lower dependence on bile salt emulsification and chylomicron formation than long-chain triglycerides (45). The released MCFAs are absorbed predominantly in their non-esterified form through the intestinal epithelium and transported directly to the liver via the portal vein. Once in the liver, they readily enter the mitochondria and undergo rapid β-oxidation catalyzed by medium-chain acyl-CoA dehydrogenase and long-chain acyl-CoA dehydrogenase, leading to the formation of ketone bodies that serve as a rapid energy source for the liver, skeletal muscle, and other tissues (46, 47). These features indicate that CO rich in medium-chain triglycerides is more rapidly digested and metabolized than oils dominated by LCFAs. At the same time, it is worth noting that CO is deficient in essential fatty acids. Linoleic acid (C18:2 n-6) and α-linolenic acid (C18:3 n-3) are typically present at levels below 2% (31, 48). Consequently, the use of CO as a primary dietary fat source may lead to inadequate essential fatty acid intake unless appropriately balanced by other lipid sources. In summary, the physicochemical properties of CO are closely associated with its high content of MCFAs, which underpin its oxidative stability, rapid digestion and absorption, and distinctive metabolic fate in animals. Importantly, this situation is further exacerbated in ruminants. The already limited amounts of C18 unsaturated fatty acids in CO will undergo extensive biohydrogenation by rumen microbes, converting them into saturated fatty acids before reaching the lower gut. Consequently, the use of CO as a primary dietary fat source may lead to inadequate essential fatty acid intake, particularly in ruminants, unless appropriately balanced by other lipid sources.

## 3 Safety of CO

Coconut oil (CO) is widely used in human food, traditional medicine, and as a feed additive in animal nutrition. In humans, moderate consumption of CO does not appear to induce significant acute or chronic toxicity. However, at high intake levels, its high SFA content has been shown to increase circulating low-density lipoprotein cholesterol (LDL-C), reflecting elevated apolipoprotein B (apoB) concentrations and an increased lipoprotein burden in the bloodstream. These lipoproteins can infiltrate the arterial intima, bind to extracellular matrix components such as proteoglycans, and subsequently undergo oxidative or enzymatic modification. This process triggers endothelial activation and inflammatory cell recruitment, leading to lipid uptake by macrophages and smooth muscle cells, foam cell formation, and fatty streak development, which may progress into atherosclerotic plaques characterized by necrotic lipid cores and fibrous caps, ultimately increasing cardiovascular disease risk (49, 50). A meta-analysis of 16 randomized controlled trials reported that CO consumption increased LDL-C concentrations by 10.47 mg/dL compared with non-tropical vegetable oils, while having no significant effect on circulating triglyceride (TG) levels, suggesting that moderate intake may be acceptable in individuals with a low baseline risk of cardiovascular disease (51). Similarly, a systematic review of 21 randomized controlled trials concluded that CO significantly increased total cholesterol (TC) and LDL-C levels to a greater extent than cis-unsaturated vegetable oils (52). Accordingly, CO intake should comply with established dietary guidelines for saturated fat, generally not exceeding 10% of total energy intake (48, 53). Nevertheless, evidence also indicates that moderate CO consumption may confer certain health benefits, including reductions in blood pressure and blood glucose levels, attenuation of cellular oxidative stress, and potential neuroprotective effects (14, 54).

When applied as an animal feed additive, the antimicrobial properties and high energy efficiency of CO have prompted interest in its use as a partial substitute for conventional lipid sources in functional energy fats, particularly in livestock, poultry, and aquaculture systems. In broiler chickens, dietary supplementation with 1.0 or 1.5 mL of CO has been shown to improve growth performance without inducing hepatic or renal damage, while enhancing antioxidant status. In contrast, inclusion levels up to 2.5 mL negatively affected feed conversion ratio, likely due to excessive caprylic acid intake (55). In weaned piglets, the combined use of CO and palm oil has been reported to improve intestinal morphology and barrier integrity, increase the abundance of *Lactobacillus* in the cecum and *Bifidobacterium* in the colon, and thereby support intestinal health (56, 57). In aquaculture species, Wang et al. demonstrated that CO could completely replace fish oil in the diet of orange-spotted grouper without compromising growth performance. However, high inclusion levels of CO (70 and 100% replacement of fish oil) markedly reduced the dietary n-3/n-6 polyunsaturated fatty acid (PUFA) ratio. This altered fatty-acid balance promoted greater utilization of MCFAs for hepatic energy metabolism, resulting in increased deposition of docosahexaenoic acid (DHA) in the liver, enhanced lipid accumulation, and a reduction in the nutritional value of fish flesh (58). Additionally, microbiological studies have shown that CO microencapsulated powder can enhance digestive enzyme activity and feed efficiency without inducing physiological abnormalities (59). In ruminants, moderate inclusion of CO maintains normal hepatic function while significantly modulating lipid metabolism. For example, CO supplementation has been shown to increase serum TC, high-density lipoprotein cholesterol (HDL-C), and LDL-C in cattle (60, 61). In addition, dose-dependent increases in serum TG and non-esterified fatty acids (NEFA) were observed in goat kids, reflecting the rapid absorption of MCFAs (23). Along with these biochemical changes, CO also affects hormonal profiles. Shi et al. (23) reported a linear increase in serum growth hormone (GH) concentrations with CO supplementation, which was associated with improved growth performance. Therefore, although CO is physiologically safe for ruminants at appropriate inclusion levels, its pronounced effects on lipid and hormonal metabolism require careful dosage management to prevent potential metabolic overload.

Overall, the safety of CO application across species is closely linked to the metabolic advantages of MCFAs. When used at appropriate inclusion levels, CO can be considered a safe and non-toxic lipid source; however, excessive supplementation may adversely affect lipid metabolism and product quality, underscoring the importance of dose optimization in both human nutrition and animal production systems.

## 4 Effects of CO on rumen microbiota

The rumen is a highly complex anaerobic microbial ecosystem that harbors a diverse consortium of microorganisms, including bacteria, protozoa, fungi, and archaea (62, 63). These microbial populations coexist in a tightly regulated symbiotic network, collectively driving feed fermentation and converting dietary substrates into microbial biomass and fermentation end-products, most notably volatile fatty acids (VFAs), which serve as the primary energy source for the host animal (64, 65). In parallel, CH_4_ production represents an integral component of rumen energy metabolism and redox balance, albeit one that is energetically inefficient from the perspective of the host (66).

**Figure 2:**
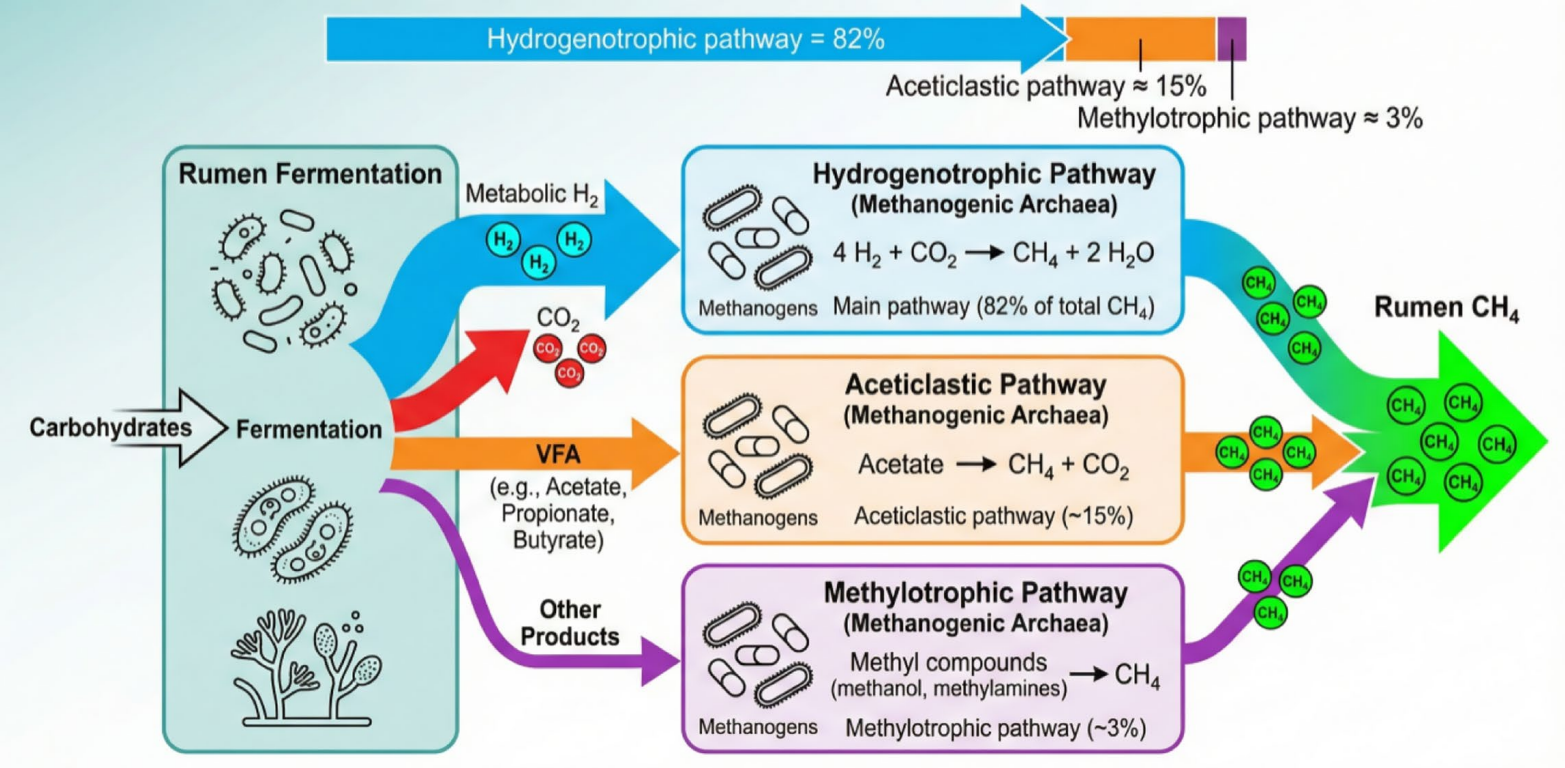
Major pathways of ruminal CH_4_ production in ruminants. The schematic illustrates the breakdown of dietary carbohydrates during rumen fermentation, generating metabolic H_2_, CO_2_, VFAs, and other intermediate products. Subsequently, methanogenic archaea utilize these substrates to produce methane via three principal pathways: the dominant hydrogenotrophic pathway, the aceticlastic pathway, and the methylotrophic pathway [based on concepts from (8, 69, 70)].

As illustrated in Figure 2, ruminal methanogenesis proceeds through three principal pathways: hydrogenotrophic methanogenesis, acetoclastic methanogenesis, and methylotrophic methanogenesis (67, 68). Among these, the hydrogenotrophic pathway is by far the dominant route, accounting for approximately 82% of total ruminal CH_4_ production (69). During carbohydrate fermentation, bacteria, protozoa, and anaerobic fungi generate metabolic H_2_ and CO_2_ as intermediate products (70). Methanogenic archaea subsequently utilize H₂ as an electron donor to reduce CO₂, which serves as the terminal electron acceptor, according to the stoichiometric reaction: 4H₂ + CO₂ → CH_4_ + 2H₂O (8). This reduction of CO₂ to CH_4_ via the hydrogenotrophic pathway plays a crucial role in maintaining ruminal redox balance by preventing the accumulation of excess H_2_, which would otherwise inhibit microbial fermentation processes (71, 72). At the same time, the conversion of CO₂ into CH_4_ contributes to carbon flux regulation within the rumen ecosystem and, to some extent, supports overall functional stability of rumen fermentation (70, 73). Nonetheless, this process inevitably leads to a loss of dietary energy for the host and the emission of a potent greenhouse gas. Previous studies have demonstrated that targeted interventions capable of modifying H_2_ availability or utilization—such as dietary lipid supplementation or manipulation of ruminal CO₂ dynamics—can markedly influence the abundance and activity of rumen protozoa and methanogenic archaea. As a consequence, such changes redirect H_2_ metabolism toward alternative sinks, alter VFA profiles, and ultimately reshape rumen fermentation patterns. The specific mechanisms by which CO modulates rumen microbial communities and methanogenic pathways will be discussed in detail in the following sections.

### 4.1 Effects of CO on rumen protozoa and archaea

The stable anaerobic environment of the rumen provides an optimal habitat for protozoa, which constitute the second most abundant microbial group after bacteria and can account for more than half of the total ruminal microbial biomass. These rumen protozoa, predominantly ciliates, exist in close symbiosis with methanogenic archaea. Their principal functions include protein degradation, predation on bacteria, and the transfer of metabolic H_2_ to associated methanogens. Methanogenic archaea, in turn, synthesize adenosine triphosphate (ATP) by establishing an electrochemical gradient through electron transfer reactions during methanogenesis, with CH_4_ representing the terminal metabolic product (74, 75). Archaea of the genus *Methanobrevibacter* perform the main work (76).

**Figure 3:**
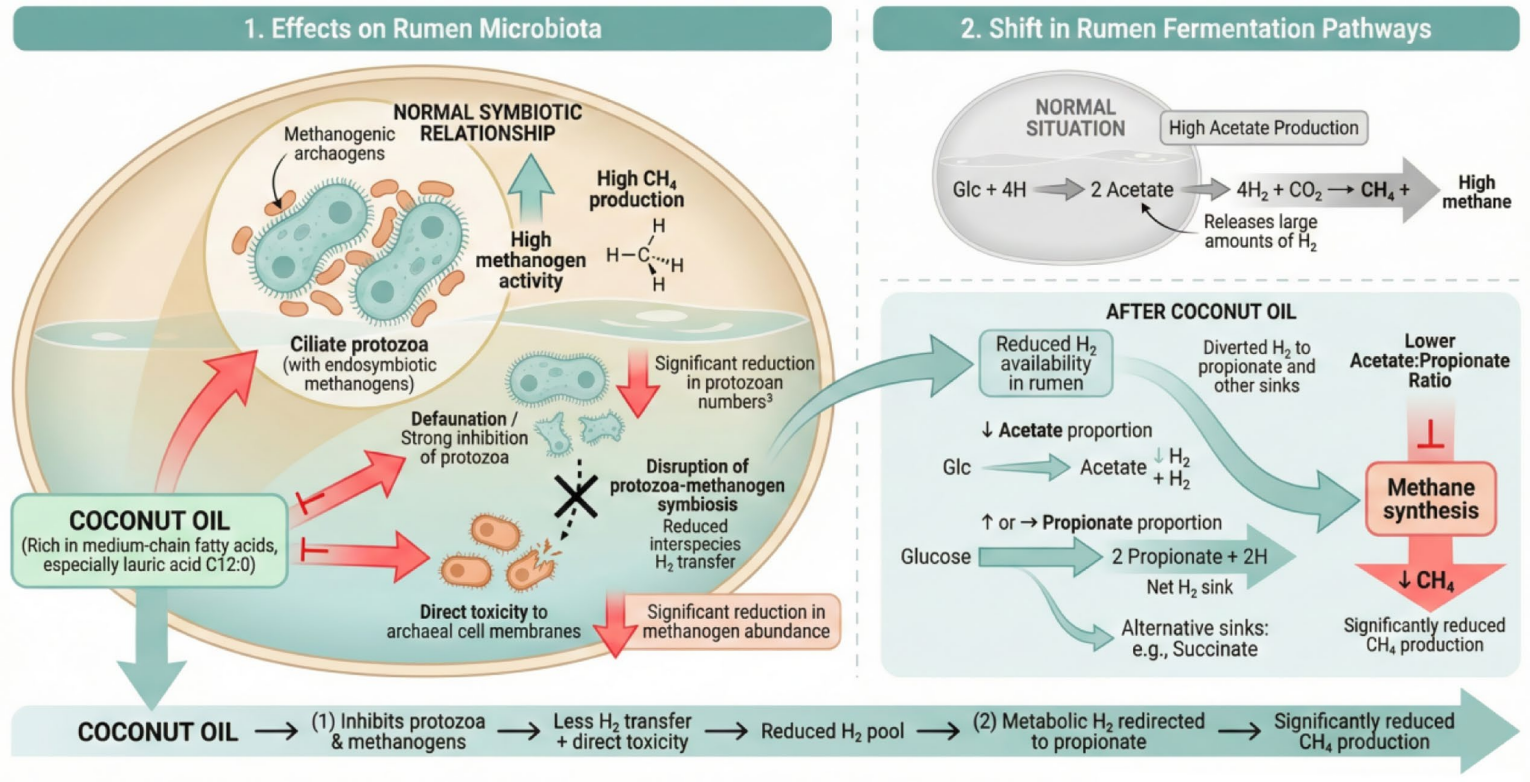
Mechanism of CO in suppressing ruminant CH_4_: microbial and fermentation pathways. Panel 1 illustrates its direct inhibitory effects on rumen microbiota, specifically disrupting the protozoa-methanogen symbiosis and causing direct toxicity to archaeal cell membranes. Panel 2 depicts the resulting shift in rumen fermentation pathways, where reduced hydrogen availability and a lower acetate-to-propionate ratio significantly suppress methane synthesis [Based on concepts from (20, 23, 25, 77, 78)].

One of the most prominent biological effects of CO is its inhibitory action on rumen protozoa and methanogenic archaea, as illustrated in Figure 3. Numerous studies have demonstrated that dietary supplementation with CO significantly reduces the abundance of ruminal ciliated protozoa and methanogenic archaea in both *in vitro* and *in vivo* systems (20, 23, 77, 78). This inhibitory effect is primarily attributed to the rapid hydrolysis of CO triglycerides by lipolytic rumen bacteria, which releases free MCFAs and SFAs that function as the principal bioactive effector molecules. At the cellular level, free fatty acids, characterized by hydrophobic carbon chains and weakly acidic carboxyl head groups, predominantly exist in their undissociated form under ruminal pH conditions. These molecules readily partition into the lipid bilayer of protozoal and archaeal cell membranes, where they form mixed lipid phases with membrane phospholipids and proteins. This interaction disrupts membrane organization and fluidity, increases membrane permeability, and causes leakage of intracellular components. Concomitantly, the dissipation of the transmembrane proton (or sodium) electrochemical gradient induces an “uncoupling” effect on microbial energy metabolism, ultimately leading to reduced cellular activity, cell lysis, and death, thereby exerting a pronounced defaunating effect (79, 80). At the microbial community level, a reduction in protozoal populations exerts both direct and indirect effects on methanogenesis. Protozoa serve as physical and metabolic niches for methanogenic archaea, harboring them on their cell surfaces and within intracellular compartments. As a result, protozoal suppression directly decreases the abundance of associated methanogens.

Furthermore, protozoa are major producers of metabolic H_2_ during fermentation; thus, their decline reduces H_2_ availability for hydrogenotrophic methanogenesis. This substrate limitation further suppresses the metabolic activity of methanogenic archaea that rely on H₂ as an electron donor (81–84). Experimental evidence strongly supports these mechanisms. An *in vitro* study reported that supplementation with 3% CO reduced protozoal density by approximately 70%, increased propionate concentration by about 15%, and decreased CH_4_ production by nearly 40% (85). Consistently, another *in vitro* investigation observed a reduction exceeding 50% in the diversity of ciliate-associated methanogenic archaea following CO supplementation (77). *In vivo*, Faciola and Broderick (25) demonstrated that dietary inclusion of 3% CO decreased rumen protozoal density by approximately 40% and significantly reduced the acetate-to-propionate ratio (A:P). These findings indicate a close association between CO-induced shifts in rumen microbial communities and alterations in fermentation patterns, characterized by reduced acetate production and increased or unchanged propionate formation, ultimately lowering the A:P (77, 86). A reduction in the A:P reflects a redirection of metabolic H_2_ away from methanogenesis toward alternative H_2_ sinks, particularly propionate synthesis, thereby contributing to CH_4_ mitigation (77). Nevertheless, the inclusion level of CO must be carefully controlled, as excessive supplementation may also inhibit fiber-degrading bacteria and impair overall rumen fermentation, leading to reduced nutrient digestibility and compromised animal performance (77, 87). These observations underscore the importance of dose optimization in practical feeding strategies to balance CH_4_ mitigation efficacy with rumen function and productive outcomes.

### 4.2 Effects of CO on rumen bacteria

**Figure 4:**
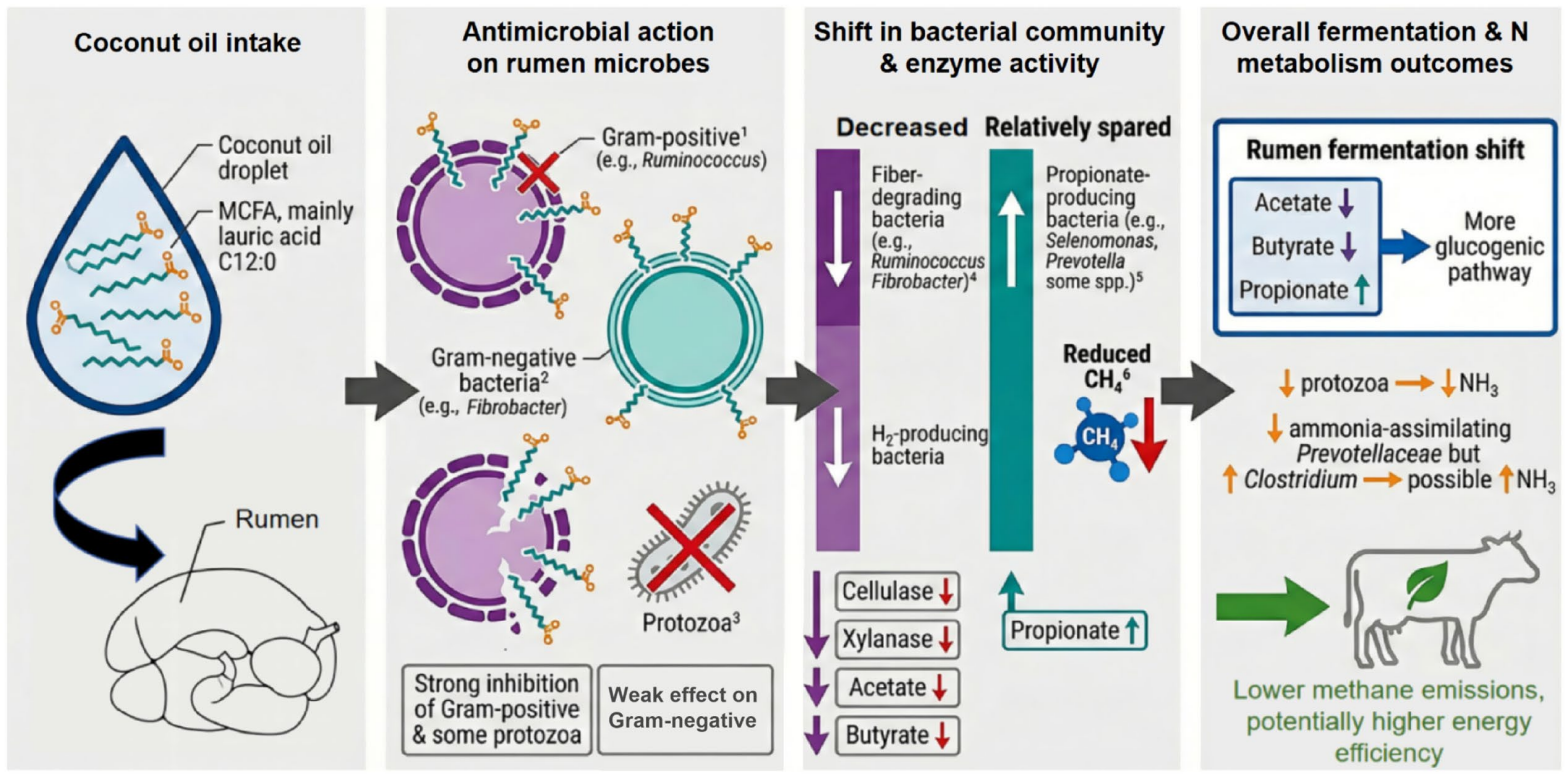
Mechanism of action of CO on rumen bacteria. This schematic demonstrates the selective inhibition of Gram-positive bacteria and protozoa by MCFAs. This antimicrobial action alters the bacterial community structure and enzyme activities, ultimately shifting rumen fermentation toward a more glucogenic pathway characterized by increased propionate production and lower methane emissions [based on concepts from (20, 23, 79, 84, 88)].

Coconut oil (CO) is rich in MCFAs, particularly lauric acid, which exhibit pronounced antibacterial properties, as illustrated in Figure 4. These fatty acids can insert into and disrupt bacterial cell membranes, impairing membrane fluidity and integrity, reducing electron transport efficiency, and dissipating the transmembrane proton gradient. As a result, bacterial cells are unable to maintain normal electrochemical potential across the membrane, which compromises ATP synthesis by ATP synthase and ultimately reduces bacterial metabolic activity. This inhibitory effect is especially pronounced in Gram-positive bacteria (88, 89). Although Gram-negative bacteria possess an outer membrane that provides a partial protective barrier, hydrophobic fatty acids are still capable of penetrating and disturbing the lipid components of this outer membrane. This leads to increased membrane permeability, disruption of transmembrane electrochemical gradients, and inhibition of bacterial growth, albeit to a lesser extent than observed for Gram-positive bacteria (79). Within the rumen environment, such membrane-disruptive effects reduce the abundance of key cellulolytic and H_2_-producing bacteria, including *Ruminococcus albus* and *Fibrobacter succinogenes* (23, 90). Hence, cellulolytic enzyme activity declines, resulting in reduced production of fermentation end-products such as acetate and butyrate. In contrast, CO exerts a comparatively weaker inhibitory effect on propionate-producing bacteria, such as members of the genus *Selenomonas*. This differential sensitivity among bacterial groups shifts rumen fermentation toward increased propionate formation (23), thereby lowering the A:P. Such a shift favors alternative H_2_ sinks and reduces H_2_ availability for methanogenesis.

Lipid hydrolysis and biohydrogenation in the rumen are primarily mediated by bacterial genera such as *Butyrivibrio* and *Anaerovibrio*, which include both Gram-positive and Gram-negative species. Consistent with the membrane-disruptive effects described above, CO supplementation has been shown to inhibit bacterial populations involved in biohydrogenation, thereby weakening this process (23, 26). As a result, a greater proportion of unhydrogenated PUFAs can escape ruminal metabolism, reach the hindgut, and be absorbed, indirectly increasing PUFA deposition in animal-derived products (61). Notably, a study in dairy calves reported that replacing milk fat with CO did not significantly alter overall rumen bacterial diversity or the relative abundance of dominant phyla (61). One plausible explanation is that the fermentative function of the calf rumen during early life is due to factors such as the esophageal groove reflex and low solid feed intake (91). In addition, the effects of CO on ruminal nitrogen metabolism remain inconsistent across studies. Some reports have associated CO supplementation with reduced ruminal ammonia nitrogen concentrations, attributed to decreased protozoal abundance. Because protozoa engulf bacteria and promote protein hydrolysis and deamination, their reduction limits bacterial protein degradation and ammonia release (20, 25). Conversely, other studies have shown that CO inhibits ammonia-assimilating bacteria, such as members of the family *Prevotellaceae*, while increasing the relative abundance of ammonia-producing *Clostridia* (from 28.4 to 41.5%), potentially leading to elevated ruminal ammonia nitrogen concentrations (84).

Overall, even in cases where significant changes in rumen bacterial diversity indices are not detected (61, 84), CO supplementation can still modify the composition and functional capacity of the rumen bacterial community. These changes tend to shift fermentation patterns toward greater energetic efficiency and lower CH_4_ emissions, with a larger proportion of dietary substrate energy being converted into propionate rather than lost as CH_4_ (20, 23). Collectively, these findings highlight the potential of CO as a natural feed additive to regulate rumen fermentation, mitigate CH_4_ emissions, and improve feed energy utilization.

### 4.3 Effects of CO on rumen fungi

Anaerobic fungi represent a key functional group involved in cellulose degradation within the rumen ecosystem. Through the development of extensive hyphae and rhizoids, these fungi can penetrate deep into plant cell walls, creating entry points that facilitate bacterial colonization and subsequent intracellular degradation of plant tissues. Moreover, rumen anaerobic fungi possess one of the most comprehensive repertoires of plant cell wall–degrading enzymes, including cellulases, hemicellulases, and specialized glycoside hydrolase families (e.g., GH5, GH6, GH48), which enable the efficient breakdown of highly recalcitrant cellulose structures (92, 93). Given the direct antimicrobial activity of CO and its high content of MCFAs, CO supplementation may exert inhibitory effects on rumen anaerobic fungi. A study conducted in Tibetan sheep demonstrated that high-dose CO supplementation (12 g/d) markedly reduced rumen fungal abundance by 85–95%, while simultaneously decreasing the populations of key cellulolytic bacteria, such as *Fibrobacter succinogenes*, by 50–98% (94). These findings indicate that CO-induced suppression of fungi may occur alongside broader inhibitory effects on fiber-degrading microbial consortia.

The mechanisms underlying fungal inhibition by CO are likely multifactorial. First, MCFAs may directly interact with fungal cell membranes in a manner analogous to their effects on bacteria and protozoa, disrupting membrane permeability, dissipating transmembrane electrochemical gradients, and impairing energy metabolism. Such disruptions may be particularly detrimental during the fungal colonization phase, leading to reduced zoospore density and suppressed fungal growth. Second, the physical presence of dietary lipids in the rumen may coat feed particles, thereby hindering microbial attachment and limiting access of fungi to fibrous substrates (26, 93, 95). Consistent with these mechanisms, reductions in rumen fungal populations following CO supplementation are often accompanied by decreases in fiber digestibility. For example, some *in vitro* studies have reported a significant decline in the digestibility of true dry matter (DM) fermented in the rumen following CO addition. However, other *in vitro* experiments have shown that although CO supplementation reduced fungal abundance in rumen fluid, the effect did not reach statistical significance (96). These inconsistent outcomes likely reflect inherent differences between *in vivo* and *in vitro* experimental systems, including microbial adaptation, substrate availability, and the absence of host-mediated regulatory processes *in vitro*. Alterations in the rumen fungal community can substantially affect fiber degradation–related metabolic pathways, leading to reduced production of fibrous fermentation end-products such as acetate. This reduction may, in turn, lower H_2_ partial pressure and favor alternative H_2_ utilization pathways, particularly increased bacterial propionate formation, resulting in decreased acetate production and elevated propionate concentrations (97).

Overall, although anaerobic fungi play a crucial role in the degradation of cellulose and hemicellulose in the rumen, current knowledge regarding the effects of CO on rumen fungal communities remains limited. Accordingly, further studies are required to elucidate the dose-dependent responses of anaerobic fungi to CO supplementation and to clarify the specific mechanisms through which CO influences fungal activity and its interactions with other rumen microorganisms.

## 5 Effects of CO on rumen fermentation

As illustrated in Figure 3, CO as a lipid source rich in MCFAs, reduces the abundance of rumen protozoa and certain cellulolytic bacteria. Therefore, CO supplementation can mildly to moderately inhibit rumen fermentation and alter VFA profiles, thereby contributing to the mitigation of enteric CH_4_ production. In *in vitro* gas production experiments, CO supplementation increased ruminal pH, significantly reduced total volatile fatty acid (TVFA) concentration, decreased the molar proportion of acetate and the A:P, and increased the molar proportion of propionate. These effects were dose-dependent and became more pronounced with increasing levels of CO inclusion. Simultaneously, protozoal populations, cellulolytic bacterial abundance, and cellulolytic enzyme activities were markedly inhibited, indicating that high doses of CO substantially weakened the intensity of cellulose fermentation while effectively reducing CH_4_ emissions (85, 98). Consistent results have been observed *in vivo*. In a dose-gradient study involving Hainan black goats supplemented with 0, 4, 6, and 8 g CO/goat/day, ruminal pH increased linearly, whereas the molar proportions of TVFA, acetate, and A:P decreased linearly, mirroring the trends observed *in vitro*. However, excessive CO supplementation also led to excessive suppression of rumen fermentation and negatively affected animal production performance (23). In contrast, in Rideau Arcott sheep, CO supplementation reduced TVFA concentration and A:P without adversely affecting production performance, possibly because only a single, moderate supplementation level was applied and excessive doses were avoided (99). Similar fermentation responses have been reported in dairy cows. Although ruminal pH was not significantly altered, CO supplementation consistently reduced the concentrations of TVFA, acetate, and butyrate, while propionate concentration remained unchanged or increased, resulting in a lower A:P (20, 25).

Collectively, these findings indicate that MCFAs in CO exhibit selective inhibitory effects on rumen protozoa, methanogens, and certain fiber-degrading bacteria. Through these mechanisms, CO suppresses acetate and butyrate production while relatively enhancing propionate formation, thereby redirecting metabolic H_2_ away from methanogenesis and reducing CH_4_ production (26, 100). More broadly, these responses are consistent with the well-established effects of high-fat diets on rumen fermentation, which are typically characterized by an increased molar proportion of propionate, a decreased proportion of acetate, and a reduced A:P (101–103). Such shifts are associated with enhanced flux through the succinate pathway of carbohydrate metabolism and increased utilization of H_2_ for propionate synthesis (104, 105), thereby limiting H_2_ availability for CH_4_ formation. In summary, moderate inclusion levels of CO exert relatively minor effects on overall rumen fermentation patterns while achieving substantial reductions in CH_4_ emissions. In contrast, excessive CO supplementation induces more pronounced alterations in rumen fermentation, which may compromise dietary energy utilization and animal production performance. These findings highlight the importance of optimizing CO dosage to balance CH_4_ mitigation efficacy with rumen function and productive efficiency.

## 6 Applications and prospects of CO in ruminant production

Coconut oil (CO) as a plant-derived lipid source, has been explored as a feed additive in ruminant production to modulate rumen fermentation and mitigate enteric CH_4_ emissions. However, a broad survey of the literature indicates that much of the foundational work on CO was conducted more than a decade ago, whereas more recent research increasingly emphasizes multi-component strategies that combine plant extracts and oils to achieve synergistic effects (106). In this context, a key objective of this review is to systematically integrate earlier evidence on CO in ruminants, thereby providing baseline data and mechanistic insights to inform future development of multi-nutrient, multi-component CH_4_ mitigation strategies.

### 6.1 Application of CO in ruminants

The application of CO in ruminants is summarized in Table 1. Owing to its high content of MCFAs, particularly lauric acid and myristic acid, CO has been widely evaluated as a functional fat source in ruminant diets. Existing *in vivo* studies include calves, bulls, dairy cows, beef cattle, sheep, goats, and buffalo, and primarily assess the integrated effects of CO supplementation on growth and lactation performance, rumen fermentation, CH_4_ emissions, gut microbiota, and overall health.

**Table 1:** Application of CO in ruminants.

Animals	Age	Weight	Dose	Experiment duration	Results (compared to the control group)	References
Hainan Black Goats	10 d	2.1 ± 0.05 kg	6 g/d	90 d	↑ Growth performance. ↓Rumen TVFAs, acetic acid, A:P and total protozoan count.	(23)
Tibetan sheep	330 d	25 ± 5 kg	12 g/d	28 d	↑ *Ruminococcus flavefaciens*. ↓ DMI, Methanogens, fungi, and *Fibrobacter succinogenes*, CH_4_ emissions.	(94)
Rideau Arcott sheep	112 d	31.5 ± 1.97 kg	25 g/kg (concentrate)	60 d	— Growth performance. ↓ TVFAs, number of methanogens and protozoa, *Fibrobacter succinogenes*, CH_4_ emissions.	(99)
Malpura lambs	15 d	——	50 g/kg (concentrate)	145 d	— Carcass quality. ↑ ADG, EE digestibility. ↓ DMI, OM and NDF digestibility, retained N, FCR, rumen protozoa, TVFAs, and NH_3_-N.	(24)
Goat kids	——	——	40 g/kg (DM; pregnant goats) and 1.8 mL/d (goat kids)	217 d	During coconut oil supplementation: ↓ early infant formula intake, ADG, TVFAs, acetic acid and A:P, total bacteria and methanogen abundance, methanogen mcrA expression. Stop coconut oil supplementation, — CH_4_ emissions, VFA concentration, and microbiome.	(111)
Hybrid beef cattle	661 ± 89 d	474 ± 29 kg	250 g/d	93 d	↑ ADG, Fatty acid methyl esters, lauric acid, and myristic acid in muscle. ↓ Rumen protozoa, CH_4_ production.	(21)
Bull	——	——	35 g/kg	84 d	— Archaea population. ↑ TVFAs, propionic acid. ↓ Acetic acid, butyric acid, A:P, and protozoa population.	(86)
Holstein calf	7 d	42.8 ± 4.4 kg	125 g/kg (Alternatives for fat in milk replacer)	56 d	— DMI, ADG, rumen bacterial community richness and diversity, dominant phyla and genera. ↑ Serum TC, HDL-C, LDL-C, and VLDL-C.	(61)
White Fulani cattle	——	138 ± 2.21 kg	150 g/d	84 d	— DMI. ↑ Serum TC, HDL-C and LDL-C. ↓ ADG.	(60)
Calf	6–12 months	——	2%(DMI)	127 d	↑ DMI, ADG. ↓ CH_4_ emissions.	(107)
Holstein cows	——	682 ± 43.9 kg	530 g/d	63 d	—DMI, milk production and milk composition. ↓ Rumen ammonia concentration, rumen protozoa abundance and CH_4_ production.	(20)
Holstein cows	——	——	2.7%(DM)	35 d	↓ DMI, OM and NDF digestibility, Milk production, CH_4_ production.	(87)
Holstein cows	——	618 ± 80 kg	500 g/d	42 d	↑ Milk Nitrogen, MCFAs (C12:0 and C14:0) and total trans fatty acids in milk. ↓ DMI and NDF digestibility, urine nitrogen, rumen protozoa	(22)
Holstein cows	——	621 ± 60 kg (multiparous); 545 ± 62 kg (primiparous)	3%(DM)	84 d	— DMI, NDF and ADF digestibility, milk yield and milk composition. ↑ Propionic acid. ↓TVFAs, acetic acid, A:P, rumen ammonia, branched-chain VFAs, milk BUN, blood BUN, and protozoa.	(25)
Holstein cows	——	——(primiparous)	5%(DM; with Rumensin)	126 d	↑ Propionic acid. ↓ DMI, NDF and ADF digestibility, TVFAs, acetic acid, butyric acid, A:P, rumen protozoa, milk yield, milk fat percentage.	(109)
Swamp buffalo	3 years	446 ± 66 kg	7% (concentrate)	84 d	↑ Propionic acid, starch hydrolysis and protein hydrolysis bacterial count. ↓ DMI, OM, NDF and ADF digestibility, TVFAs, acetic acid, A:P, rumen protozoa, *Fibrobacter succinogenes* population and CH_4_ production.	(108)
Swamp buffalo	1 year	200.5 ± 9.5 kg	6% (concentrate; with sunflower oil)	150 d	— Carcass quality. ↑ Acetic acid, A:P. ↓ DMI, ADG, NH_3_-N, BUN, protozoa and bacterial count.	(110)

——, No; —, No effect; ↑, Increase; ↓ Decrease. TVFAs: Total volatile fatty acids; A:P: Acetic acid to propionic acid ratio; OM: Organic matter; NDF: Neutral detergent fibers; ADF: Acid detergent fibers; FCR: Feed conversion ratio; ADG: Average daily gain; DMI: Dry matter intake; DM: Dry matter; EE: Ether extract; TC: Total cholesterol; HDL-C: High-density lipoprotein cholesterol; LDL-C: Low-density lipoprotein cholesterol; VLDL-C: Very-low-density lipoprotein cholesterol; NH_3_-N: Ammonia nitrogen; BUN: Blood urea nitrogen (2.1 ± 0.05 (mean ± standard error of the mean [SEM])).

Regarding growth performance, several studies have reported that low-to-moderate CO inclusion can improve average daily gain and feed conversion ratio to varying degrees, consistent with enhanced energy utilization and more favorable nutrient partitioning (21, 23, 24, 94, 107). In contrast, other studies have shown that when CO constitutes a high proportion of DMI (generally >3% of dietary DM), DM digestibility and growth rate may decrease (20, 22, 87, 108–110). These adverse responses are likely attributable to the suppression of fiber-degrading microorganisms and fiber degradation, reduced rumen microbial activity and fermentation intensity, and potential increases in rumen digesta retention time, which collectively can depress feed intake. Overall, these findings suggest a clear upper limit for practical CO use and highlight the need to optimize supplementation levels under production conditions.

In lactating cows, the effects of CO and MCFA-rich fat preparations on milk yield remain inconsistent across studies. While some experiments reported no significant change in milk yield, many studies observed reduced milk production, largely associated with decreased DMI and reduced neutral detergent fiber (NDF) digestibility (20, 87, 109). Meanwhile, CO supplementation frequently remodels milk fatty acid profiles, increasing the proportion of medium-chain SFAs while decreasing LCFAs, particularly certain UFAs, thereby altering the nutritional value and physical properties of milk fat (20, 22, 25, 87). Under high inclusion levels, decreased milk fat percentage and a higher risk of negative energy balance have also been reported; thus, CO may be better considered a nutritional tool for modifying milk fat composition rather than a strategy solely aimed at maximizing milk yield (20, 25).

One of the most extensively investigated applications of CO is the regulation of rumen microbiota and fermentation to suppress methanogenesis. The selective antimicrobial activity of MCFAs is central to CO-driven changes in rumen ecology and CH_4_ formation. As shown in Table 1, most *in vivo* studies have confirmed that CO reduces protozoal abundance and enteric CH_4_ emissions. Correspondingly, rumen VFA profiles often shift toward a higher molar proportion of propionate and a lower proportion of acetate and/or butyrate, resulting in a lower A:P. This pattern reflects improved H_2_ utilization efficiency and reduced H_2_ availability for methanogenesis, consistent with CH_4_ mitigation and enhanced energy recovery. Despite these findings, when CO inclusion increases beyond moderate levels, some studies have reported reductions in TVFA concentration and fiber digestibility, along with variable changes in rumen pH, suggesting that excessive MCFAs can inhibit fiber-degrading microorganisms and thereby compromise fiber utilization and energy supply (20, 89, 94). In addition, some reports suggest that CO, potentially via antimicrobial and surfactant-like properties, may support intestinal barrier function and inhibit colonization by opportunistic pathogens. Nevertheless, the current evidence is largely derived from short-term indicators or indirect inference, and comprehensive validation using metagenomic and metabolomic approaches remains limited (23, 94, 111). With respect to health and immunity, available *in vivo* evidence remains relatively scarce but suggests potential benefits. Under pasture-based conditions or heat stress, moderate CO supplementation has been associated with improved blood lipid profiles, reduced inflammatory markers, and enhanced antioxidant capacity and non-specific immune indices, which may relate to udder health, maintenance of body condition, or postpartum recovery (23, 60, 107, 111).

### 6.2 Future prospects

**Figure 5:**
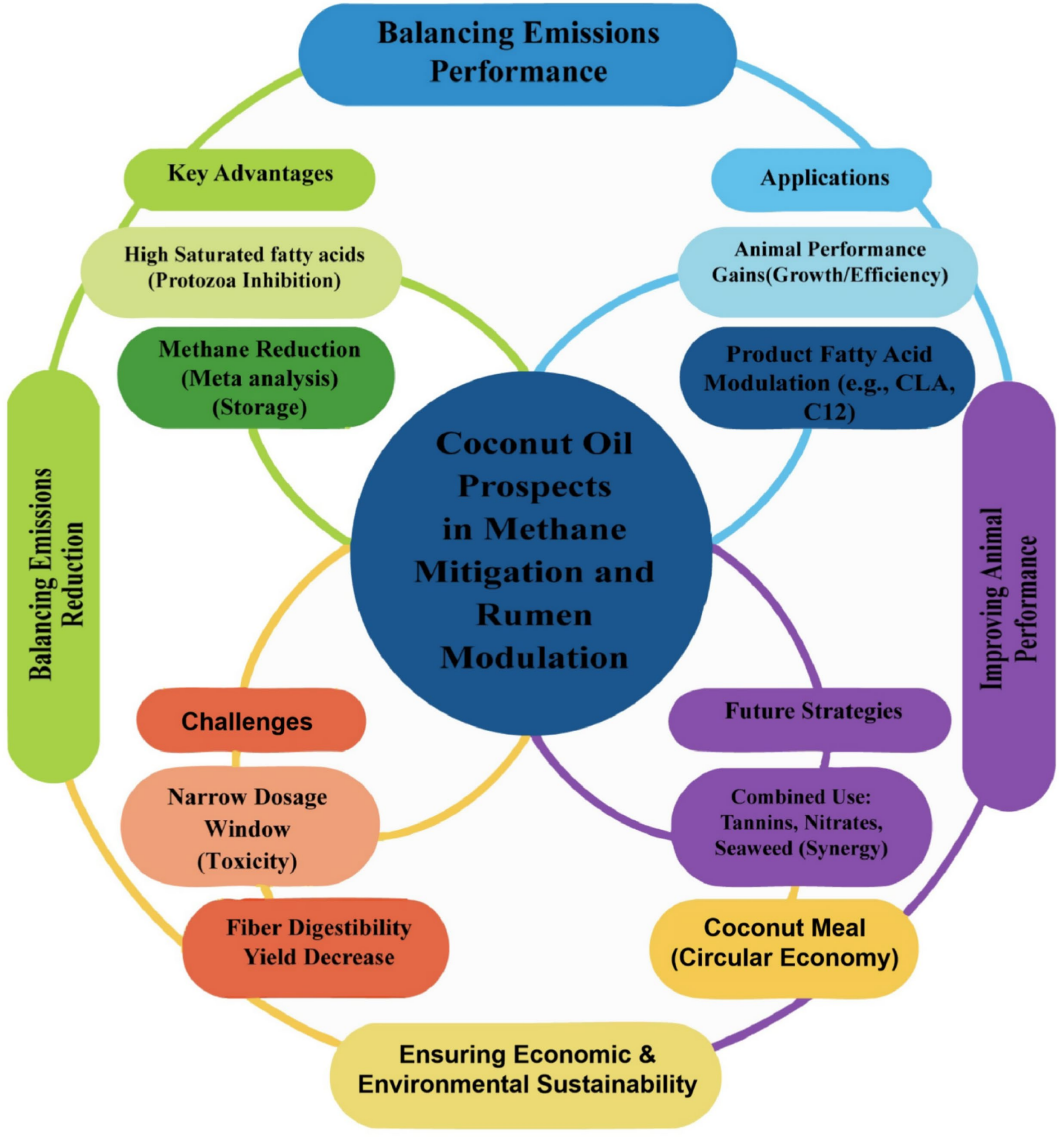
Prospects for the application of coconut oil in ruminant animal livestock farming.

The preceding sections have summarized evidence from *in vivo* and *in vitro* studies evaluating CO in ruminant systems. Its primary value lies in CH_4_ mitigation and the regulation of rumen ecology within the context of sustainable ruminant production. Nevertheless, practical implementation remains constrained by challenges related to dose optimization and broader sustainability considerations (Figure 5).

Compared with other vegetable oils, CO contains an exceptionally high proportion of SFAs; this compositional feature is associated with stronger inhibitory activity against rumen protozoa. In parallel, the high saturation of CO confers excellent oxidative stability, which supports the feasibility of long-term storage and practical handling of CO under farming conditions. Mechanistically, the distinctive fatty acid profile of CO underpins its capacity to suppress rumen protozoa and methanogenic archaea, thereby reducing CH_4_ production. As summarized in Table 1, when supplemented at appropriate levels, CO commonly brings out the advantages of propionic acid and improves apparent energy utilization efficiency. Nonetheless, these findings suggest that although CO is a promising intervention, its effective “dose window” may be relatively narrow, and inappropriate use can amplify adverse impacts on rumen function and animal performance, a dose threshold of <5% of dietary DM is recommended. A recent meta-analysis of 41 *in vitro* and *in vivo* studies confirms this, showing that in *in vivo* experiments, the effective inhibitory dose of MCFA addition is approximately 4% of dietary DM, beyond this amount, methane inhibition tends to exhibit diminishing returns, and further increases may negatively affect digestibility (19). Therefore, future implementation should aim to minimize CH_4_ emissions while balancing productive performance and the nutritional quality of animal-derived products. Ideally, the specific dosage should be adjusted based on factors such as animal type, growth stage, and intended use.

It should be noted that CO could be positioned as one component of integrated CH_4_ mitigation strategies. In sheep, chestnut tannins and CO have been reported to independently and additively reduce enteric CH_4_ emissions and protozoal abundance without impairing growth performance, suggesting that MCFAs may interact beneficially with plant secondary metabolites such as tannins (99). In beef and dairy cattle, combining dietary nitrates with lipids or 3-nitrooxypropanol has produced additive or partially additive reductions in CH_4_ emissions, while also emphasizing the need to monitor potential trade-offs involving DMI and animal performance (112–114). Moreover, seaweed-derived halogenated compounds and polysaccharides have been shown to mitigate CH_4_ through distinct microbial and biochemical mechanisms (115–118). These pathways are conceptually complementary to MCFA-driven suppression of methanogens and protozoa; however, the efficacy and safety of combining CO with seaweed-based interventions have not yet been evaluated *in vivo* and warrant targeted investigation.

Notably, evidence regarding CO-driven rumen modulation and CH_4_ mitigation may, to some extent, be translatable to coconut meal, a byproduct of CO extraction. Mechanical pressing typically leaves coconut meal with approximately 7% residual oil, provides roughly 19–24% crude protein, and contains high levels of NDF, while retaining a fatty acid signature enriched in MCFAs, particularly lauric acid and myristic acid (119, 120). This suggests that coconut meal could function as a diluted carrier of MCFAs, potentially eliciting CH_4_ suppression and microbial remodeling effects similar to those of CO (77). Consistent with this notion, a systematic review and meta-analysis indicated that MCFAs from different sources can reduce CH_4_ production and modulate rumen fermentation patterns under both *in vitro* and *in vivo* conditions (19). Meanwhile, the high fiber content and imbalanced amino acid profile of coconut meal limit its use in monogastric species (119, 121, 122), making it more suitable for ruminant feeding. This approach also aligns with the broader goals of value-added utilization of oil-processing byproducts, the development of circular bioeconomy systems, and environmental sustainability.

## 7 Conclusion

Coconut oil (CO) is rich in MCFAs and SFAs, which can serve dual functions in ruminants by modulating rumen ecology and mitigating enteric CH_4_ emissions. At appropriate inclusion levels, CO can selectively suppress rumen protozoa, methanogenic archaea, and certain fiber-degrading bacteria, thereby redirecting H_2_ metabolism, decreasing the A:P, and, in some studies, improving energy utilization and growth performance. However, excessive supplementation frequently impairs fiber digestibility, reduces DMI, and compromises lactation performance, indicating that the effective mitigation window for CO may be relatively narrow and requires optimization according to species, diet type, and production stage. Overall, evidence supporting the application of CO in ruminant systems remains limited and, in some areas, inconsistent. Future research should therefore prioritize systematic evaluation of dose-dependent responses in rumen microecology and host metabolism, identify optimal inclusion levels under practical feeding conditions, and develop application strategies that achieve CH_4_ mitigation without sacrificing productivity or product quality.
